# 

**Published:** 1854-07

**Authors:** 


Vol. I. JULY, 1854. No. 12.

### IOWA

### MEDICAL JOURNAL

CONDUCTED BY THE FACULTY OF THE

**Medical Department of the Iowa University.**

PUBLISHED MONTHLY.

TERMS--TWO DOLLARS, IN ADVANCE.

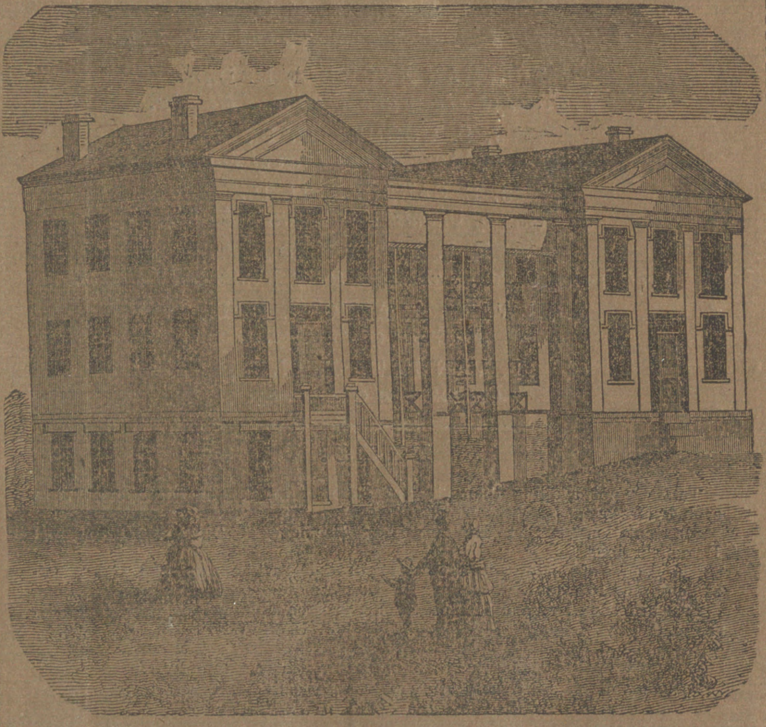

KEOKUK, IOWA:

Printed at the Whig Book and Job Office.

☞ ADDRESS ALL COMMUNICATIONS, POST-PAID, TO "MEDICAL COLLEGE, BOX 109, KEOKUK, IOWA." ☜

